# Amplification of the Sylvatic Cycle of Dengue Virus Type 2, Senegal, 1999–2000: Entomologic Findings and Epidemiologic Considerations

**DOI:** 10.3201/eid0903.020219

**Published:** 2003-03

**Authors:** Mawlouth Diallo, Yamar Ba, Amadou A. Sall, Ousmane M. Diop, Jacques A. Ndione, Mireille Mondo, Lang Girault, Christian Mathiot

**Affiliations:** * Institut Pasteur de Dakar, Dakar, Senegal; † Université Cheikh Anta Diop de Dakar, Senegal

**Keywords:** dengue virus, mosquitoes, sylvatic, Kedougoa, Senegal, research

## Abstract

After 8 years of silence, dengue virus serotype 2 (DENV-2) reemerged in southeastern Senegal in 1999. Sixty-four DENV-2 strains were isolated in 1999 and 9 strains in 2000 from mosquitoes captured in the forest gallery and surrounding villages. Isolates were obtained from previously described vectors, *Aedes furcifer*, *Ae. taylori*, *Ae. luteocephalus,* and—for the first time in Senegal—from *Ae. aegypti* and *Ae. vittatus.* A retrospective analysis of sylvatic DENV-2 outbreaks in Senegal during the last 28 years of entomologic investigations shows that amplifications are periodic, with intervening, silent intervals of 5–8 years. No correlation was found between sylvatic DENV-2 emergence and rainfall amount. For sylvatic DENV-2 vectors, rainfall seems to particularly affect virus amplification that occurs at the end of the rainy season, from October to November. Data obtained from investigation of preimaginal (i.e., nonadult) mosquitoes suggest a secondary transmission cycle involving mosquitoes other than those identified previously as vectors.

Dengue is a viral disease transmitted by mosquitoes and caused by four viral serotypes (dengue virus serotypes 1–4 [DENV 1–4]) belonging to the genus *Flavivirus* of the *Flaviviridae* family. In terms of illness and death, dengue is the most important viral disease transmitted to humans by mosquitoes (*1*). This virus is distributed nearly worldwide and represents a serious public health problem in Southeast Asia, the Caribbean, the Pacific Islands, and Latin America. While in Asia and the Americas human-to-human transmission by mosquitoes is the current form of virus circulation, in West Africa sylvatic circulation is predominant. In Africa, the existence of dengue dates back to 1956,when a retrospective serosurvey confirmed that a dengue epidemic occurred in Durban, South Africa, in 1926–1927 (*2*). Additional evidence was obtained in the 1960s, when DENV-1 and DENV-2 were isolated for the first time from human samples in Nigeria (*3*).

In Senegal, evidence of dengue virus circulation was obtained when DENV-2 was isolated for the first time in 1970 from human blood (*4*). After this isolation, an entomologic surveillance program was undertaken in Senegal. By that time, in light of the yellow fever virus transmission cycle established by Haddow (*5*), much interest was being shown in sylvatic cycles of arboviruses in general. The main objective of this program was to identify the vectors and describe the sylvatic cycle of dengue virus transmission. Results obtained from these studies identified several DENV-2 epizootics through periodic amplification of the sylvatic cycle in Kedougou, Senegal (*6*–*8*). Although DENV 1–4 were isolated incidentally in Senegal from humans, only DENV-2 was shown to be circulating regularly in mosquito, human, and monkey populations with a sylvatic focus in Kedougou (*9*,*10*).

After 8 years of silence, DENV-2 reemerged in 1999. In this paper we analyze data obtained during this 1999–2000 epizootic in light of climatic changes and vector ecology. We also discuss new insights in terms of the maintenance and emergence mechanisms of DENV-2.

## Materials and Methods

### Study Sites and Calendar of Investigations

Our study area was located in the southeastern part of Senegal (12°11′W, 12°33′N) in Kedougou, a department (the first-level administrative subdivision of the region) named after the town Kedougou, which is surrounded by an area of savannah and forest. In this paper the expression Kedougou area refers to the Savannah and forest galleries area, where most of our study took place. The population is essentially rural. The Kedougou area, which belongs to the Sudan-Guinean climate, is located at 1,200–1,300 mm isohyets; the area is part of the rainiest Senegalese region. The rainy season generally lasts from May/June to October/November, with maximum rainfall generally recorded in August or September. We conducted entomologic investigations in the Kedougou area in June, October, and November 1999. This schedule for site visits was based on our previous experience in the field and had two objectives: 1) to investigate vertical transmission of arboviruses with the emergence of nulliparous adult mosquitoes at the beginning of a rainy season after an arbovirus amplification, and 2) to isolate the maximum amount of virus at the end of the rainy season, the period of maximum arbovirus amplification. After results of virus isolation from mosquitoes showed evidence of DENV-2 circulation in 1999, we conducted intensive mosquito collections during the rainy season in June, August, September, October, and November 2000 .

### Mosquito Sampling

**Figure 1:**
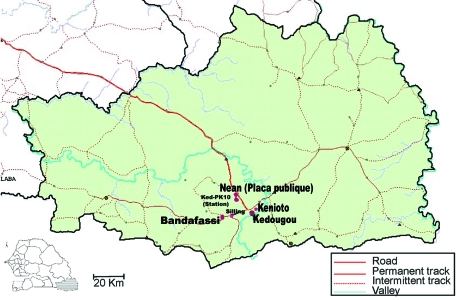
Map of the Kedougou area, Senegal, showing geographic position of villages and forest gallery where dengue virus serotype 2 vectors were collected.

Because many arboviruses of medical and veterinary interest circulate in the study area, we used a variety of sampling methods to collect a wide range of mosquito species. Mosquitoes were then sampled by using human landing catches, CDC light traps with or without CO_2,_ and animal bait traps. However, for collection of dengue vectors, we captured mosquitoes exclusively by human landing collections, using persons vaccinated against yellow fever virus and taking malaria chemoprophylaxis. These captures occurred between 5:30 p.m. and 8:30 p.m. in the forest gallery (located 10 km from Kedougou) and the villages of Ngari, Silling, Bandafassi, and Kénioto (Figure 1). The ecologic characteristics of the area have been described (*7*,*11*). Twenty-four human volunteers participated each evening, including 18 persons in the forest gallery and 6 in one village. In the forest gallery, mosquito catches were performed at the ground level and in the canopy. Five platforms 6–9 m high served as capture sites in the canopy. Captured mosquitoes were frozen and then sorted on a chill table by using identification keys established by Edwards (*12*), Ferrara et al. (*13*), Huang (*14*), and Jupp (*15*). Mosquitoes were sorted into monospecific pools and frozen in liquid nitrogen for virus isolation attempts.

To verify virus maintenance in the field by vertical transmission (after evidence of DENV-2 circulation was obtained in 1999), wild mosquito breeding sites were investigated. This investigation was undertaken during the dry season (outside of the adult mosquito’s activity season), in February 2000, when all sylvatic mosquito breeding sites were dry. Tree holes were scraped with spoons and knives to collect *Aedes* eggs, known to be resistant to desiccation. Samples from these tree holes were stored in plastic boxes and flooded in the laboratory for egg hatching. Larvae were reared to the adult stage, then frozen, identified, and pooled by species for virus isolation attempts.

### Virus Isolations

Virus isolations were performed on AP61 (*Aedes pseudoscutellaris*) mosquito cells lines, as described by Digoutte et al. (*16*). Virus was identified by using immunofluorescence with a specific immune ascitic fluid and confirmed by complement fixation or neutralization tests.

### Monkey Serum Sampling

After initial results of virus isolation from mosquitoes showed evidence of DENV-2 circulation, serologic investigations on monkeys were undertaken January 31–February 6, 2000. Blood samples were collected from each wild monkey captured with flexible nets in the forest gallery. Briefly, animals were immobilized with ketamine HCl (Merial, Lyon, France), 10–15 mg/kg of body weight, injected intramuscularly. Blood was collected by femoral venipuncture into a heparinized vacuum tube, and gender was determined by inspection. Dental casts, morphologic measurements, and features relating to reproductive status (i.e., nipple length, scrotal pigmentation) were used to assign age classes of the animals. Each was tagged and released in the wild. In the field laboratory, heparinized blood was centrifuged for 15 minutes at 2,000 rpm, and the plasma was removed and stored in liquid nitrogen until testing. Serum samples were tested for DENV-2 immunoglobulin M (IgM)/IgG antibodies by using the enzyme-linked immunosorbent assay, as described (*17*).

### Rainfall Analysis

Rainfall fluctuations from 1972 to 1999 were retrospectively analyzed with respect to 1961–1990 mean rainfall (the normal level, as defined by the World Meteorological Organization) and correlated to DENV-2 emergence in the same period. Anomalies were calculated by subtracting the recorded seasonal rainfall during May to October in each year from the seasonal rainfall mean (or normal) from 1961 to 1990. Rainfall data were provided by Agence pour la Sécurité de la Navigation Aérienne, Dakar, Senegal. Only rainy season months were taken into account in the analysis.

### Data Analysis

Several entomologic indices were estimated: 1) the true infection rate (estimated number of dengue virus–positive mosquitoes per 100 mosquitoes tested) by using the methods of Chiang and Reeves (*18*) and Walter and others (*19*); 2) and the entomologic inoculation rate (number of infected mosquito bites per human per evening). Rates obtained were compared by using the chi-square test with p <0.05 considered significant.

## Results

A total of 24,747 mosquitoes belonging to six genera and 55 species were collected by using all sampling methods in 1999. Table 1 lists the number of pools and infection rates of mosquito species infected with DENV-2. Sixty-four DENV-2 strains were isolated and distributed as follows: *Ae*. *furcifer* (35 strains), *Ae*. *taylori (*11 strains), *Ae*. *luteocephalus* (16 strains), and *Ae*. *aegypti* (2 strains) captured in October and November. From mosquitoes collected in June, no DENV-2 strain was isolated.

**Table 1:** Mosquitoes collected and dengue virus serotype 2 infection rates of potential vectors, Kedougou, 1999

Species	June		October				November				Total			
	No. specimens captured	(No. positive/ total pools)	No. specimens captured	(No. positive/ total pools)	True infection rate^a^		No. specimens captured	(No. positive/ total pools)	True infection rate^a^		No. specimens captured	(No. positive/ total pools)	True infection rate^a^	
*Aedes furcifer* male	2	(0/1)	–	(0/0)	–		27	(0/6)	–	–	29	(0/7)	-	-
*Ae. furcifer* female	1,132	(0/36)	1,998	(10/56)	1.72	[0.34]	1,398	(25/41)	2.84	[0.58]	4,528	(35/133)	0.91	[0.15]
*Ae. taylori* male	21	(0/7)	2	(0/1)	–	–	32	(0/5)	–	–	55	(0/13)	-	-
*Ae. taylori* female	358	(0/12)	122	(1/5)	0.92	[0.92]	543	(10/19)	2.63	[0.85]	1,023	(11/36)	1.28	[0.39]
*Ae. luteocephalus* female	1,064	(0/35)	682	(8/22)	1.45	[0.52]	392	(8/13)	3.06	[1.17]	2,138	(16/70)	0.84	[0.21]
*Ae. aegypti* female	54	(0/8)	6	(0/2)	–	–	15	(2/4)	17	[11.57]	75	(2/14)	2.74	[1.97]
Other mosquitoes^b^	5,549	(0/197)	3,366	(0/165)	–	–	7,984	(0/276)	–	–	24,747	(0/638)	-	-

^a^True infection rate (ratio in percentage of the number of dengue virus serotype 2 strains isolated/total number of mosquitoes captured); [standard error]; –, no data obtained. ^b^Other mosquitoes: *Anopheles coustani, An. ziemanni, An. brohieri, An. brunnipes, An. domicola, An. flavicosta, An. funestus, An. gambiae s.l., An. hancoki, An. maculipalpis, An. nili, An. pharoensis, An. pretoriensis, An. rufipes, An. squamosus, Ae. argenteopunctatus, Ae. centropunctatus, Ae. dalzieli, Ae. fowleri, Ae. hirsutus, Ae. minutus, Ae. ochraceus, Ae. vexans, Ae. vittatus, Ae; mcintoshi, Ae. cozi, Ae. metallicus, Ae. neoafricanus, Ae. unilineatus, Culex annulioris, Cx. antennatus, Cx. bitaeniorhynchus, Cx. decens, Cx. duttoni, Cx. ethiopicus, Cx. neavei, Cx. perfuscus, Cx. poicilipes, Cx. tritaeniorhynchus, Cx. cinereus, Cx. nebulosus, Cx. tigripes, Mansonia africana, Ma. uniformis, Mimomyia* *mediolineata, Mi. mimomyiaformis, Mi. plumosa, Uranotaenia fusca, Ur. mashonaensis, Ur. balfouri,* and *Ur. mayeri.*

The highest mean infection rates were obtained from *Ae. aegypti* (2.74%), followed by *Ae. taylori* (1.28%). However, mean infection rates of species did not differ significantly (p=0.34). Infection rates showed temporal and spatial variations. Most virus strains were isolated from mosquitoes captured in November. Infection rates observed during that month were often higher than those obtained in October, but only *Ae. furcifer* showed a significant difference between these 2 months (p<0.001). Table 2 shows the spatial distribution of biting, infection, and inoculation rates for mosquito species associated with DENV-2. In the forest gallery, *Ae. furcifer, Ae. taylori,* and *Ae. luteocephalus* were very aggressive, whereas *Ae. aegypti* displayed weak biting activity. The highest biting rate was obtained from *Ae. furcifer* (average 4.16 bites per person per hour). The lowest biting rate was obtained from *Ae. aegypti* (0.08 bite per person per hour). The maximum rate for *Ae*. *furcifer* was obtained in October (5.87 bites per person per hour). For *Ae. luteocephalus* and *Ae. taylori,* the maximum rate occurred in June (3.28 bites per person per hour) and November (1.64 bite per person per hour), respectively. In the villages, only *Ae. furcifer* had significant activity, with a maximum of 8.75 bites per person per hour in Ngari. When collections from Silling, Bandafassi, and Ngari were compared, the highest biting rates of the species regularly collected in the villages were obtained in October.

**Table 2:** Temporal and spatial distribution of vector activity and infection in Kedougou, 1999^a^

Species	Nos. and rates^a^	Mosquitos captured in forest gallery			Ngari			Silling			Bandafassi			Kénioto
		June	Oct	Nov	June	Oct	Nov	June	Oct	Nov	June	Oct	Nov	Nov
*Aedes furcifer* male	n	1	–	8	–	1	7	1	–	–	–	–	3	2
*Ae. furcifer* female	n	1,053	1,586	1,204	2	315	110	37	33	23	21	66	50	18
	BR	3.25	5.87	3.65	0.16	8.75	3.66	2.05	2.5	1.27	1.75	5.5	4.17	1
	TIR	–	0.51	2.99	–	–	2.85	–	3.13	–	–	16.12	–	5.55
	EIR	–	0.08	0.32	–	–	0.31	–	0.23	–	–	2.67	–	0.65
*Ae. luteocephalus* female	n	1,063	682	390	–		2	1	–		–		–	-
	BR	3.28	2.52	1.18	–	–	0.07	0.05	–		–	–	–	-
	TIR	-	1.45	3.09	–	–	–	–		–	–	–	–	-
	EIR	–	0.11	0.11	–	–			–	–	–		–	-
*Ae. aegypti* female	n	45	5	15	5	1	–	4	–	–	1	–	–	-
	BR	0.13	0.45	0.04	0.42	0.03	–	0.22			0.08	–	–	-
	TIR	–	–	17	–	–	–	–	–	–	–	–	–	-
	EIR	–	–	0.02	–	–	–	–	–	–	–	–	–	-
*Ae. taylori* male	n	21	2	32	–	–	–	–	–	–	–	–	–	-
*Ae. taylori* female	n	358	122	543	–	–	–	–	–	–	–	–	–	-
	BR	1.1	0.45	1.64	–	–	–	–	–		–		–	-
	TIR	-	0.92	2.63	–	–	–	–	–	–	–	–	–	-
	EIR	-	0.01	0.13	–	–	–	–	–	–	–	–	–	-

^a^ n, number of specimens captured; BR, biting rate (number of mosquitoes captured per human per hour); Oct, October; Nov, November; TIR, true infection rate (estimated number of positive mosquitoes per 100 mosquitoes tested according to Chiang and Reeves [18] and Walter et al. [19]); EIR, entomologic inoculation rate (number of infected mosquito bites per human per evening); –, no data obtained.

Our results (Table 2) show that DENV-2 circulated in the forest gallery and all the sampled villages. Indeed, among the 64 DENV-2 strains isolated, 58 were isolated from mosquitoes caught in the forest gallery and 6 from those caught in the villages: Ngari (2 strains), Silling (1 strain), Bandafassi (2 strains), and Kenioto (1 strain). In the forest gallery, except for *Ae. furcifer,* which exhibited significantly higher infection rates in November than in October (p<0.001), infection rates for all species were comparable in October and November. In the Kedougou area, the highest infection rate was obtained from *Ae. aegypti* (17%). However, the highest inoculation rate was obtained from *Ae. furcifer,* which was estimated to be responsible for at least two infected bites per person per week in November. *Ae. furcifer* was also the only mosquito infected with DENV-2 in the villages. The highest entomologic inoculation rate was obtained in Bandafassi, where a person might receive at least two infectious bites each evening.

A total of 2,423 adult mosquitoes belonging to the genus *Aedes* and the subgenera *Albuginosus, Stegomyia, Aedimorphus, Diceromyia,* and *Finlaya* emerged from the tree hole samples collected during the dry season in 2000. *Ae. aegypti*, *Ae. bomeliae,* and *Ae. luteocephalus* were the most common species. Out of 68 pools, no DENV-2 was isolated (Table 3).

**Table 3:** Mosquito adults emerging from tree hole samples

Mosquito species	No. of specimens	No. of pools	DENV-2^a^ isolations
*Aedes (Diceromyia) taylori*	75	2	0
*Ae. (Diceromyia) furcifer*	1	1	0
*Ae. (Aedimorphus) dalzieli*	24	2	0
*Ae. (Albuginosus) stokesi*	27	1	0
*Ae. (Finlaya) longipalpis*	16	2	0
*Ae. (Stegomyia) aegypti*	1,305	26	0
*Ae. (Stegomyia) luteocephalus*	331	15	0
*Ae. (Stegomyia) bromeliae*	510	16	0
*Ae. (Stegomyia) unilineatus*	134	3	0
Total	2,423	68	0

^a^DENV-2, dengue virus serotype 2.

During the 2000 rainy season, out of 31,521 mosquitoes collected in the same locations, nine DENV-2 strains were isolated, all associated with circulation of yellow fever virus. Isolations were obtained from female mosquitoes including *Ae. furcifer* (one strain), *Ae. taylori* (two strains), *Ae. luteocephalus* (two strains), *Ae. aegypti* (one strain), *Ae*. *vittatus* (two strains); isolations were obtained from males of *Ae. furcifer* (two strains) captured in the forest gallery. Isolations were obtained in August (three strains), October (five strains), and November (one strain).

DENV-2 IgG was detected in nonhuman primate blood samples, but no evidence of recent infection (IgM antibody) was obtained. Of a total of 17 African green monkeys (*Chlorocebus sabaeus*) collected, 8 juveniles (<4 years of age) and 9 adults were captured in a forest gallery near Ngari. In all samples, serologic test results were negative for DENV-2 IgM antibody. A seroprevalence of 58% for DENV-2 IgG antibodies was detected; no significant difference (p=0.09) was detected according to age (77% in adult and 37% in juvenile monkeys).

**Figure 2:**
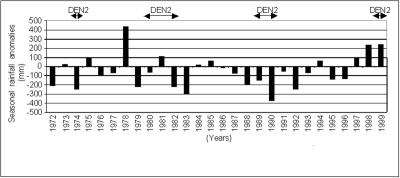
Seasonal rainfall anomalies in Kedougou, Senegal, 1972–1999. Anomalies were calculated by subtracting the recorded seasonal rainfall during May to October in each year from the seasonal rainfall mean (normal), 1961–1990.

The years 1999–2000 were characterized by a rainfall surplus compared to the 1960–1999 seasonal mean rainfall. Retrospective analysis showed no clear relationship between dengue emergence and rainfall anomalies. DENV-2 amplifications were detected during periods of heavy rainfall as well as during periods of low precipitation (Figure 2).

## Discussion

After 8 years of silence, DENV-2 reemerged in a sylvatic cycle in the Kedougou area in 1999. With new isolations, the number of DENV-2 strains isolated from mosquitoes in Senegal reached 350. Except for *Ae. aegypti* and *Ae. vittatus,* which were associated with DENV-2 for the first time in Senegal, all strains were obtained from vectors previously described for the DENV-2 sylvatic cycle in West Africa and belonging to the subgenus *Diceromyia* (*Ae. furcifer and Ae. taylori*) and *Stegomyia* (*Ae. luteocephalus*) (*20*–*22*). Population density of vector species and their spatial dynamics permitted us to assess a potential role in the transmission cycle for each infected mosquito species. *Ae. taylori* and *Ae. luteocephalus,* because of their scarcity in villages, may have a role limited to the forest gallery. However, *Ae. furcifer* may contribute to both sylvatic transmission and virus dissemination from the forest zone to human habitats since this species is the only infected one abundant in the domestic environment. *Ae.* *aegypti*, the principal DENV-2 epidemic vector worldwide, while very scarce in our catches, was more abundant in the forest gallery than in the villages. *Ae. aegypti*’s low rate of biting humans may reflect the zoophilic tendency of this species, which probably exists in the area only in its sylvatic form, *Ae. aegypti formosus* (*11*), which uses tree holes as breeding sites. (By contrast, the domestic form, *Ae. aegypti aegypti*, preferentially colonizes artificial water containers.) However, the low entomologic inoculation rates of *Ae. aegypti* in sylvatic areas suggests its limited role in sylvatic amplification cycles of DENV-2 despite its high infection rates. Although *Ae. vittatus* was infected with DENV-2 in our study, its role in dengue transmission has never been demonstrated. Further studies are needed to determine its involvement in the sylvatic cycle.

Sylvatic amplifications of DENV-2 have previously been observed in Senegal in 1974, 1980–1982, and 1989–1990 (*6*–*8*), defining periods of occurrence with silent intervals of 5–8 years (*23*). This periodicity of occurrence shows the similarity between DENV-2 and yellow fever virus, which share the same vectors, vertebrate hosts, and ecologic niche (*8*). Moreover, this pattern of occurrence shows that DENV-2 is maintained in the Kedougou area by a still-unknown mechanism, although two have been hypothesized: vertical transmission of the virus or its maintenance through a secondary cycle. Vertical transmission is the most probable hypothesis since it has been supported by laboratory and field evidence for *Ae. aegypti* (*24*,*25*) and *Ae. taylori* (*6*). The isolation from male *Ae. furcifer* during this investigation is the first field evidence of vertical transmission of DENV-2 by this species in Kedougou and reinforces this hypothesis. Our investigation of tree holes also supports the hypothesis of a secondary transmission cycle involving mosquito species other than those identified as vectors. These investigations showed high representation of species considered scarce or absent, which was the case for *Ae.* *longipalpis* belonging to the *Finlaya* subgenus, a forest/enzootic vector of dengue in Asia; *Ae. bromeliae*, one of the major yellow fever virus vectors in East Africa; and *Ae.* *stockesi* (*1*,*14*,*26*). The role of these species may have been overlooked because of biased sampling methods for mosquito captures. Further research about the existence of a secondary cycle is needed.

In DENV-2 sylvatic circulation, vertebrate hosts may serve either as amplifiers or reservoirs. Unfortunately, data obtained in this study from monkey serum samples were not conclusive about their role during amplification because of IgG cross-reactions between flaviviruses (*27*). Thus, the role of monkeys in sylvatic cycle of DENV-2 remains unresolved. Extensive investigations are needed to assess the role of vertebrate hosts in the amplification and maintenance of DENV-2 in natural conditions through herd immunity, turnover of susceptible vertebrates hosts, or persistent infection (*28*,*29*).

Analysis of rainfall amount and distribution did not show any correlation with sylvatic DENV-2 emergence unlike the situation with other arboviruses such as Rift Valley fever (*30*,*31*). Rainfall has an impact on two epidemiologic parameters important in arbovirus transmission: vector density, which controls the transmission level, and adult mosquito longevity, which makes transmission possible and durable. For the sylvatic DENV-2 vectors in the Kedougou area, rainfall seems to interfere, particularly with the virus amplification period (generally October–November), coinciding with the end of the rainy season. Indeed, although vector population densities are already high at the beginning of the rainy season, the virus emergence and the maximum amplification period occur only at the end of the rainy season. Probably the greater longevity of the female vectors at the end of the rainy season, attributable to low intensity of precipitation, allows them to achieve a complete extrinsic incubation of DENV-2.

DENV-2 isolations from *Ae*. *furcifer* captured in peridomestic habitats demonstrate that the virus circulates in all villages where mosquito catches were undertaken. However, no DENV-2 clinical case was recorded in the region. This finding suggests that DENV-2 is confined to the forest or, if human-mosquito-human transmission occurs, the level is low and leads to nonsymptomatic cases. Similar situations were observed during the 1981 and 1990 DENV-2 epizootics, when only a few sporadic human cases were recorded (*32*,*33*). The same situation occurred in Burkina Faso and Côte d’Ivoire (*21*,*22*,*34*), where sylvatic transmission of DENV-2 was observed without an epidemic. Only one epidemic, which occurred in Burkina Faso in 1982, has been reported in West Africa; that epidemic was suspected of being caused by the introduction of a virus strain from the Seychelles Islands (*35*). Therefore, the risk of a DENV-2 epidemic in Senegal is very low if only the sylvatic cycle is taken into account. However, a risk exists since importation of an epidemic strain in an urban area cannot be excluded, as witnessed by the epidemic in Burkina Faso in 1982.
